# Functional analysis of Ca^2+^ signalling in *Besnoitia besnoiti* tachyzoites

**DOI:** 10.1017/S0031182025101182

**Published:** 2026-01

**Authors:** Camilo Larrazabal, Daniela Grob, Zahady D. Velásquez, Carlos Hermosilla, Anja Taubert, Iván Conejeros

**Affiliations:** 1 Department of Veterinary Sciences and Public Health, Universidad Catolica de Temuco, Temuco, Chile; 2 Institute of Parasitology, Justus Liebig University Giessen, Giessen, Germany

**Keywords:** *Besnoitia besnoiti*, Ca^2+^ signalling, M-3M3FBS, Phospholipase C

## Abstract

*Besnoitia besnoiti* is an apicomplexan parasite, the causal agent of bovine besnoitiosis. This disease is characterised by cyst formation in the skin and mucosa. During early infection, fast proliferating tachyzoites invade and replicate within host endothelial cells. In non-excitable cells, extracellular signals activate the inositol-triphosphate/calcium (InsP3/Ca^2+^) pathway, which depends on phospholipase C (PLC) activation, inducing an increase in IP_3_ levels, followed by intracellular Ca^2+^ release. Despite the understanding of Ca^2+^ signalling, this process in *B. besnoiti* tachyzoites is unclear. This work aimed to study Ca^2+^ dynamics during *B. besnoiti* infection in bovine umbilical vein endothelial cells (BUVEC) and the role of the InsP_3_/Ca^2+^ pathway during *B. besnoiti* infection. Ca^2+^ dynamics during tachyzoite replication were determined in *B. besnoiti-*infected BUVEC loaded with fluo-4-AM. The role of InsP_3_/Ca^2+^ signalling for parasite invasion was evaluated by treatments with Ca^2+^ chelators (BAPTA, EGTA) or PLC inhibitors (U73122, D609). PLC activation was studied in fluo-4-loaded free tachyzoites using the PLC activator m-3M3FBS, in the presence or absence of PLC inhibitors. Current data show an infection-driven increase in total Ca^2+^ signals in *B. besnoiti*-infected BUVEC over time. BAPTA, but not EGTA, treatments of tachyzoites affected their invasion, reducing infection rates by 85.4 ± 9.3%, suggesting that intracellular Ca^2+^ sources are necessary for *B. besnoiti* invasion. In line, treatments with U73122 and D609 reduced infection rates by 79.3 ± 9.4% and 49.7 ± 8.9%, respectively, demonstrating that PLC participation is required for host cell infection. Finally, m-3M3FBS treatments induced a PLC-independent Ca^2+^ flux in *B. besnoiti* tachyzoites.

## Graphical abstract

**Figure:**
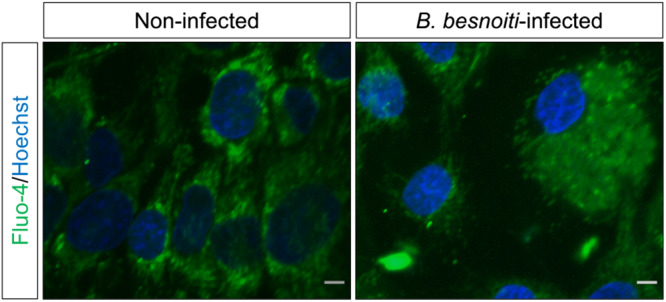

## Introduction

*Besnoitia besnoiti* is an obligate intracellular apicomplexan parasite that causes bovine besnoitiosis and affects cattle industry, besides causing detrimental effects on animal welfare. The definitive host of this coccidian parasite is still unestablished, being likely mechanically transmitted within infected herds via blood sucking insects (i.e. tabanids or stable flies), while the participation of wild ruminants as reservoirs has been proposed (Alvarez-García et al. 2013). Since 2010, cattle besnoitiosis has been considered an emerging disease in the EU (Alvarez-García et al. 2013). In contrast to other cyst-forming coccidian parasites, such as *Neospora caninum, Toxoplasma gondii* or *Sarcocystis* spp., *B. besnoiti* tissue cysts are mainly found in skin and mucosa (Alvarez-García et al. 2014; Langenmayer et al. 2015). Overall, acute bovine besnoitiosis is characterised by unspecific clinical signs, such as lethargy, anorexia, tachycardia and tachypnoea, accompanied by febrile responses of infected animals as a consequence of inflammatory reactions induced by tachyzoite proliferation (Alvarez-García et al. 2014). In this context, endothelial cells serve as major host cells for *B. besnoiti* tachyzoite proliferation *in vivo*. Respective infections induce vasculitis, thrombosis and necrosis in venules and arterioles (Langenmayer et al. 2015). Besides other cell types, proliferation of *B. besnoiti* tachyzoites can easily be performed in bovine primary endothelial cells *in vitro* (Maksimov et al. 2016; Jiménez-Meléndez et al. 2020).

The tachyzoite-based merogonic lytic cycle is a multi-step process initiated when free tachyzoites actively invade a suitable host cell, proliferate within a parasitophorous vacuole (PV) and finally egress by host cell lysis (Black and Boothroyd, 2000). On a mechanistic level, the lytic cycle of apicomplexan parasites is tightly regulated by Ca^2+^ signalling (Black and Boothroyd, 2000; Lourido and Moreno, 2015; Hortua Triana et al. 2018). Overall, cumulated evidence indicates that Ca^2+^ fluxes in apicomplexans, such as *T. gondii,* are linked to invasion, conoid extrusion, microneme discharge, intracellular proliferation and egress (Lourido and Moreno, 2015; Hortua Triana et al. 2018). In this context, apicomplexan parasites store their intracellular Ca^2+^ within organelles, including endoplasmic reticulum (ER), apicoplast and acidocalcisomes (Lourido and Moreno, 2015; Hortua Triana et al. 2018), allowing them to tightly modulate cellular responses. Physiologically, cytoplasmic Ca^2+^ acts as a coupling factor and second messenger for multiple cellular functions (Carafoli and Krebs, 2016). In general, resting cells accumulate Ca^2+^ in intracellular stores, the ER and mitochondria (Carafoli and Krebs, 2016). In non-excitable cells, such as endothelial cells, extracellular signals are propagated downstream via the activation of the inositol 1,4,5-triposphate/calcium (InsP_3_/Ca^2+^) pathway (Berridge, 2016). Activation of this pathway is initiated by the interaction of a cytoplasmic receptor with its ligand, leading to the activation of phospholipase C (PLC). PLC catalyses the hydrolysis of phosphatidylinositol 4,5-bisphosphate (PIP₂) into diacylglycerol (DAG) and inositol trisphosphate (IP₃). IP₃ then binds to its receptor (IP₃R), triggering the release of Ca^2^⁺ ions into the cytoplasm (Berridge, 2016). Besides, other Ca^2^⁺-related signalling pathways present in specific cell types can induce intracellular Ca^2^⁺ fluxes via other receptors, such as ryanodine receptors (RyR).

Nonetheless, Ca^2+^ signalling has been comparatively much less studied in apicomplexan species than in mammalian cells. Interestingly, the presence of PLC homologs in different apicomplexans, including *T. gondii* (Fang et al. 2006) and *B. besnoiti* (Strain Bb-Ger1; ToxoDB), indicates a conserved role of this enzyme. In this context, a conditional knockout of PLC in *T. gondii* tachyzoites and *Plasmodium falciparum* merozoites causes highly altered phenotypes and severe impairment of intracellular development, thereby compromising the cellular lytic cycle and *in vivo* proliferation (Bullen et al. 2016; Burda et al. 2023) and demonstrating a pivotal role of this enzyme in apicomplexan biology. Likewise, pharmacological blockage of PLC in *T. gondii* tachyzoites interferes with conoid extrusion and microneme discharge, affecting host cell invasion (Ricard et al. 1999; Del Carmen et al. 2009; Bullen et al. 2016). Even though a participation of IP_3_R- and RyR-sensitive mechanisms has been proposed for both *T. gondii* tachyzoites and *P. chaubadi*/*P. falciparum* merozoites (Passos and Garcia, 1998; Lovett et al. 2002; Alves et al. 2011), the absence of IP_3_R and ryanodine receptor homologues in apicomplexan parasites indicates significant divergences in apicomplexans.

To our best knowledge, details on Ca^2+^ signalling in *B. besnoiti* tachyzoites are currently lacking and related data almost exclusively come from the closely related and much more studied apicomplexan parasite, *T. gondii*. Interestingly, downstream Ca^2+^-dependent protein kinase (CDPK) homologues are proposed as new pharmacological targets for *B. besnoiti* infection control (Jiménez-Meléndez et al. 2017). The current work aims to study Ca^2+^ dynamics in *B. besnoiti* tachyzoites, in the context of PLC activation-related mechanisms and the role of different Ca^2+^ stores in *B. besnoiti* tachyzoites in infected primary bovine endothelial cells.

## Materials and methods

### Host cells and *Besnoitia besnoiti* tachyzoite in vitro culture

Primary bovine umbilical vein endothelial cells (BUVEC) were isolated as described elsewhere (Taubert et al. 2006). BUVEC were cultured at 37 °C and 5% CO_2_ atmosphere in modified endothelial cell growth medium (modECGM), prepared by the dilution of complete ECGM medium (PromoCell; Heidelberg, Germany) with M199 (Sigma-Aldrich; St. Louis, USA) at a 1:3 ratio. The medium was supplemented with 500 U/mL penicillin (Sigma-Aldrich), 50 μg/mL streptomycin (Sigma-Aldrich; St. Louis, USA) and 5% foetal calf serum (FCS; Biochrom; Cambridge, UK). BUVEC (Isolates= 6) were seeded into 12-well plates (Sarstedt; Nümbrecht, Germany) pre-coated with fibronectin (1:400; Sigma-Aldrich; St. Louis, USA) and cultured until confluence. BUVEC with no more than three passages were used in this study.

Tachyzoites of *B. besnoiti* (strain Bb Evora04) were propagated in Madin Darby bovine kidney cells (MDBK) in RPMI medium (Sigma-Aldrich; St. Louis, USA), supplemented with 500 U/mL penicillin and 50 μg/mL streptomycin and 5% FCS (all Sigma-Aldrich). Infected and non-infected cells were cultured at 37 °C and 5% CO_2_ atmosphere. Vital tachyzoites were collected from supernatants of infected host cells by a centrifugation step at 800 × g for 5 min, and resuspended either in modECGM or HBSS depending on the experimental setup.

### *Besnoitia besnoiti* tachyzoite treatments and infection assays

As illustrated in Table 1, for PLC inhibition-related experiments, freshly collected *B. besnoiti* tachyzoites (six biological replicates) were pre-treated for 30 min at 37 °C with the PLC inhibitor U73122 (5 µM; AdipoGen Life Sciences; San Diego, USA) or vehicle (0.02% DMSO, Sigma-Aldrich). Complementarily, given that U73122-derived Ca^2+^ fluxes are triggered by sarco/ER Ca^2+^ ATPase (SERCA) inhibition (Macmillan and McCarron, 2010), additional experiments with the PLC-specific inhibitor D609 (5 µM; Cayman; Ann Arbor, USA) were included in this study. Inhibitor-treated and untreated tachyzoites were used to infect BUVEC at a multiplicity of infection (MOI) of 8:1 in the presence of inhibitors or vehicle. This MOI is justified on the early infection time points here studied (see below). Additionally, to establish the role of intracellular or extracellular Ca^2+^ sources on *B. besnioti* infection, chelation experiments were carried out. Briefly, *B. besnoiti* tachyzoites were harvested as described above and treated with 50 µM BAPTA-AM (Invitrogen; Thermofisher; Waltham, USA) or vehicle (same as above) for 30 min at 37 °C for intracellular Ca^2+^ chelation. Excess BAPTA was removed by washing with PBS (5 min, 800 × g). Thereafter, treated and untreated *B. besnoiti* tachyzoites were used to infect BUVEC as described above in the presence or absence of 5 mM EGTA (extracellular Ca^2+^ chelator, Sigma-Aldrich; St. Louis, USA), to evaluate the effects of extracellular chelation (EGTA) or intracellular and extracellular chelation (BAPTA + EGTA). In addition, endothelial host cell infection by newly released *B. besnoiti* tachyzoites from previously infected cells was considered to establish the infection rate time points (Jiménez-Meléndez et al. 2019). Specifically, since *B. besnoiti* tachyzoite (strain BbEvora4) proliferation in BUVECs is characterised by an increase in the number of tachyzoites per PV after 12 post-infection (p.i.) (Velásquez et al. 2020), only earlier time points p.i. were evaluated to avoid proliferation-driven infection rate effects. Given that BUVEC layers exhibit a flat morphology, intracellular tachyzoites can easily be detected by phase contrast microscopy. Thus, phase contrast images were acquired at one or three h p.i. by an inverted microscope (IX81®, Olympus) equipped with a digital camera (XM10®, Olympus) for infection rate assessment. Finally, the infection rate was established as [(infected cells/total cells) × 100], from four randomly selected fields on each studied time point (1 and 3 h p.i.) as described elsewhere (Larrazabal et al. 2021b).

**Table 1.** Summary of main treatments and assays performed in this study

Compound	Supplier	Cat. number	Working concentration	Target	References
BAPTA-AM	Invitrogen; Thermofisher	B6769	50 µM	Intracellular Ca^2+^ chelator	Lovett et al. (2002)
EGTA	Sigma-Aldrich	E4378	5 mM	Extracellular Ca^2+^ chelator	Lovett et al. (2002)
Fluo4-AM	Invitrogen; Thermofisher	F14201	2.5 µM	Calcium-sensitive fluorescent probe	Larrazabal et al. (2021a; b)
m − 3M3FBS	Cayman	16867	5 µM	PLC agonist	Bae et al. (2003)
U73122	AdipoGen Life Sciences	AG-CR1 − 3698	5 µM	PLC antagonist	Bullen et al. (2016)
D609	Cayman	13307	5 µM	PLC antagonist	Ricard et al. (1999)

### Intracellular Ca^2+^ measurements

Registries of Ca^2+^ signals were obtained by loading BUVEC (Isolates = 5) or *B. besnoiti* tachyzoites (six biological replicates) with the Ca^2+^-sensitive dye fluo-4 (Invitrogen; Thermofisher; Waltham, USA) as described before (Larrazabal et al. 2021a; 2021a). Briefly, host cells or free tachyzoites were incubated for 30 min at 37 °C in HBSS in 2.5 µM fluo-4. Thereafter, excess dye was removed by PBS washing (5 min, 800 × g) and cells were suspended in HBSS. For experimentation, fluo-4-loaded *B. besnoiti* tachyzoites (2 × 10^6^ and 1 × 10^7^ tachyzoites/ml) were placed into a 96-well plate (Sarstedt; Nümbrecht, Germany) per experimental condition and then exposed to m-3M3FBS (PLC agonist, 5 µM, Cayman; Ann Arbor, USA). In this context, the concentration of m-3M3FBS used in this study was justified by the absence of previous reports in *B. besnoiti* or other apicomplexans, as well as by the cytotoxic effects on free tachyzoites observed at higher concentrations (25 µM; Supplementary Figure 1). In case of BUVEC-related Ca^2+^ quantification, cells were seeded into a 12-well plate (Sarstedt; Nümbrecht, Germany), infected with *B. besnoiti* tachyzoites (MOI 3:1), thereafter loaded with fluo-4 as described above and lysed by 0.1% Triton X-100 (Sigma-Aldrich; St. Louis, USA) treatments at 6, 12 and 24 h p.i. Spectrofluorometric analysis at an excitation wavelength of 488 nm and an emission wavelength of 530 nm was performed in an automated multiplate monochrome reader (Varioskan Flash®, Thermo Scientific). No technical replicates were carried out in Ca^2+^ flux assays.

**Figure 1.**
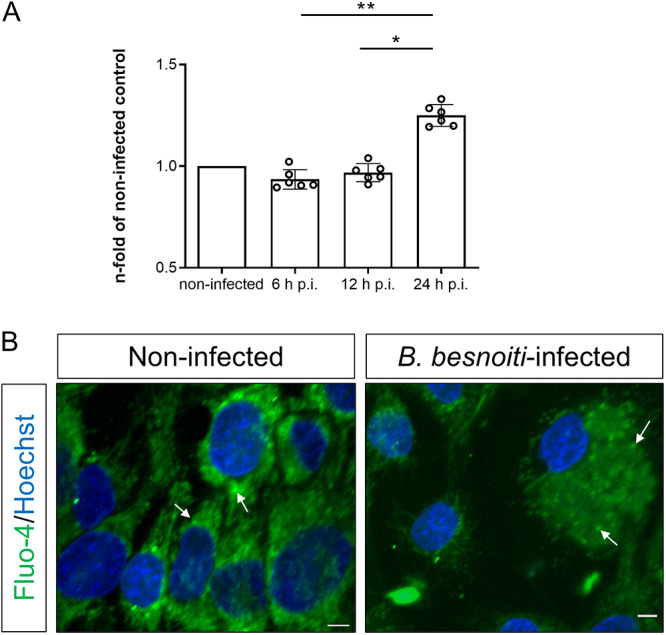
*Besnoitia besnoiti* infection increases host cellular Ca^2+^ levels. (A) Fluo-4-based Ca^2+^ signals at different hours post-infection (h p.i.) in *B. besnoiti*-infected BUVEC, expressed as fold of its respective non-infected control (B) LSCM from non-infected and *B. besnoiti*-infected BUVEC at 24 h p.i. Arrows indicate Fluo-4-derived signals located in intracellular vesicles. Size scale bars correspond to 5 μm. Bars represent means of five biological replicates ± standard deviation. *p ⩽ 0.05; **p ⩽ 0.01.

### Live cell laser scanning and 3D holotomographic microscopy

For live cell imaging, BUVEC were seeded into 25.5 × 75.5 mm tissue culture µ-dishes (Ibidi, Gräfelfing, Germany) and cultured (37ºC, 5% CO_2_) until confluence. Thereafter, freshly collected *B. besnoiti* tachyzoites were allowed to infect the monolayers using an MOI of 3:1. For Ca^2+^ flux analyses on free *B. besnoiti* tachyzoites, 1 × 10^6^ fluo-4-loaded tachyzoites were placed into 25.5 × 75.5 mm 8-well tissue culture µ-dishes (Ibidi; Gräfelfing, Germany). Ca^2+^-related spatial distribution – as mirrored by fluo-4-derived signals – was analysed in both *B. besnoiti*-infected cells and free *B. besnoiti* tachyzoites. Laser scanning confocal fluorescence microscopy (LSCM) was performed by a ReScan Confocal Microscope instrumentation (RCM 1.1 Visible, Confocal NL; Netherlands) equipped with a fixed 50 µm pinhole size and combined with a Nikon Ti2-A inverted microscope and a motorised Z-stage (DI1500, Nikon). The RCM unit was connected to a Toptica (TOPTICA Photonics SE; Germany) iChrome CLE laser with the following excitation wavelengths: 405/488/561/640 nm. Images were acquired via a sCMOS camera (PCO edge) using a CFI Plan Apochromat 60 × lambda-immersion oil objective (NA 1.4/0.13; Nikon). The system was operated by the microscope software NIS-Elements (Nikon; v 5.11). Images were acquired via a z-stack optical series with a step size of 0.4 microns. Finally, 3D live cell holotomography was performed by a 3D Cell Explorer Fluo microscope (Nanolive; Switzerland) at 60 × magnification (excitation wavelength = 520 nm, sample exposure 0.2 mW/mm^2^ and a depth of field of 30 µm) using LED as light source (CoolLED pE-300ultra; Andover, UK).

### Image processing and analysis

Infection rates were calculated from phase contrast images in an observer-blind manner. Fluorescence-based images were analysed with Fiji ImageJ v1.54 using Z-projection and merged channel plugins. In addition, live cell 3D-holotomographic images were analysed using STEVE^®^ software v.2.6 (Nanolive; Switzerland) to obtain a z-stack based on refractive index (RI) of organelles. Furthermore, digital staining was applied according to the RI of tachyzoites and inner organelles.

### Statistical analysis

For statistical analyses, GraphPad^®^ Prism 8 (version 8.4.3) software was used. Ca^2+^ flux changes over time were analysed by area under the curve (AUC) estimation during 370 s after treatment, using the first 50 s prior to stimulation as baseline. Likewise, to accurately illustrate Ca^2+^ fluxes, baseline correction was performed by subtracting from each data point the average value obtained during the first 50 sec before stimulation. Data description was carried out by presenting the arithmetic mean ± standard deviation. To compare two experimental conditions, the non-parametric statistical Mann–Whitney test was applied. In cases of three or more conditions, Kruskal–Wallis test was applied. Whenever global comparison by Kruskal–Wallis test indicated statistical significance (*p* ≤ 0.05), Dunn post-hoc multiple comparison tests were carried out to compare treated and control conditions. Outcomes of statistical tests were considered to indicate significant differences at *p ≤* 0.05 (significance level).

## Results

### Infection with Besnoitia besnoiti tachyzoites drives Ca^2+^ accumulation in host cells

To address whether *B. besnoiti* infection drives alterations in intracellular Ca^2+^ concentration, we evaluated total fluo-4-derived calcium signals in lysed *B. besnoiti* tachyzoite-infected BUVEC cultures throughout merogony. Current data showed a significant enhancement of Ca^2+^-mediated fluorescence in *B. besnoiti*-infected BUVEC layers at 24 h p.i. when compared with earlier time points (Figure 1A). Specifically, an increase to 134.01 ± 8.89% (*p* = 0.001) and to 129.28 ± 6.48% (*p* = 0.029) was detected when comparing 6 and 12 p.i. with 24 h p.i., respectively. Furthermore, live cell scanning laser confocal microscopy illustrated enhanced Ca^2+^ signals within *B. besnoiti* meronts in infected BUVEC at 24 h p.i. (Figure 1B). In non-infected BUVEC, a rather vesicular pattern of Ca^2+^-related signals was observed in the cytoplasm, whilst Ca^2+^ signals mainly accumulated in tachyzoites inside meronts in *B. besnoiti*-infected BUVEC (Figure 1B).

### Intracellular Ca^2+^ and PLC signalling governs Besnoitia besnoiti tachyzoite invasion

**Figure 2.**
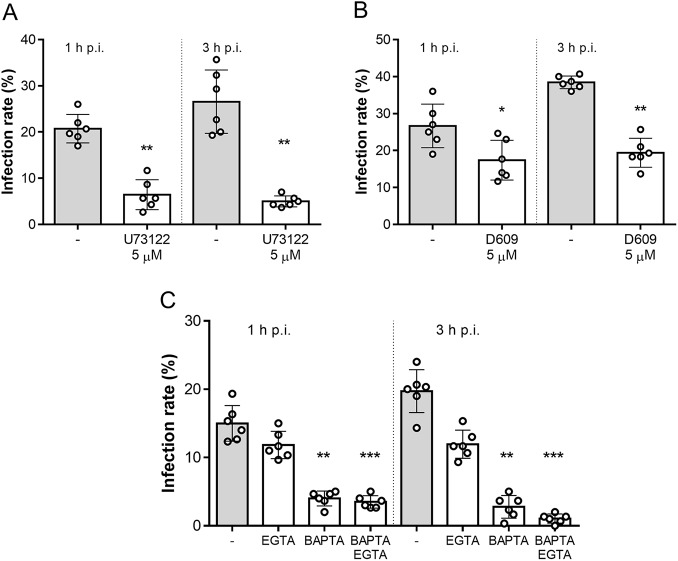
*Besnoitia besnoiti* invasion relies on PLC activity and on intracellular Ca^2+^ stores. Bar graphs illustrate infection rates at 1 and 3 h p.i. of *B. besnoiti* tachyzoites exposed to U73122 (A) D609 (B) or Ca^2+^ chelators (C). Bars represent means of six biological replicates ± standard deviation. *p ⩽ 0.05; **p ⩽ 0.01. ***p⩽ 0.001.

To test whether this mechanism also applies to *B. besnoiti*, we performed functional invasion assays using *B. besnoiti* tachyzoites pre-treated with the aminosteroid PLC blocker U73122. As shown in Figure 2A, 5 µM U73122 pre-treatments significantly reduced infection rates, compared with vehicle-treated tachyzoites, by 72.48 ± 11.23% (*p* = 0.002) and 77.71 ± 8.52% (*p* = 0.002) at 1 and 3 h p.i., respectively. In line, treatments with another PLC blocker (D609, 5 µM) also significantly blocked parasite host cell invasion as reflected by reduced infection rates at both 1 h p.i. (41.74 ± 16.94%, *p* = 0.017) and 3 h p.i. (49.26 ± 8.88%, *p* = 0.002) (Figure 2B). Additionally, we aimed to address the role of intra- and extracellular Ca^2+^ sources in tachyzoite invasion. Therefore, tachyzoites were treated with BAPTA (50 µM, intracellular Ca^2+^ chelator) or EGTA (5 mM, extracellular Ca^2+^ chelator) to block intra- and extracellular Ca^2+^ sources. In case of EGTA treatments, infection rates were only diminished by tendency [20.62 ± 7.80% (*p* = 0.49) and 37.63 ± 15.07% (*p* = 0.86) at 1 and 3 h p.i., respectively]. In contrast, BAPTA treatments strongly affected parasite invasion since infection rates were reduced by 72.53 ± 8.49% (*p* = 0.005) and 85.42 ± 15.70% (*p* = 0.003) at 1 and 3 h p.i., respectively. Finally, combinatory treatments with both BAPTA and EGTA significantly blocked parasite invasion by 76.72 ± 3.76% (*p* = 0.0001) and 94.97 ± 2.76% (*p* = 0.0007) at 1 and 3 h p.i., respectively (Figure 2C).

### Treatments with m-3M3FBS induce a fast but PLC-independent calcium flux in Besnoitia besnoti tachyzoites

**Figure 3.**
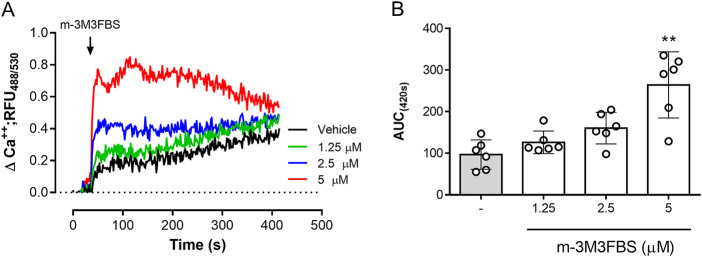
m-3M3FBS treatments induce a fast and sustained Ca^2+^ flux in *B. besnoiti* tachyzoites. (A) Fluorometric registries over time of fluo-4-loaded m-3M3FBS-treated (1.25–5 µM) *B. besnoiti* tachyzoites. (B) Bar graph showing the AUC at 420 s average ± standard deviation of six biological replicates. ** *P* ⩽ 0.01.

**Figure 4.**
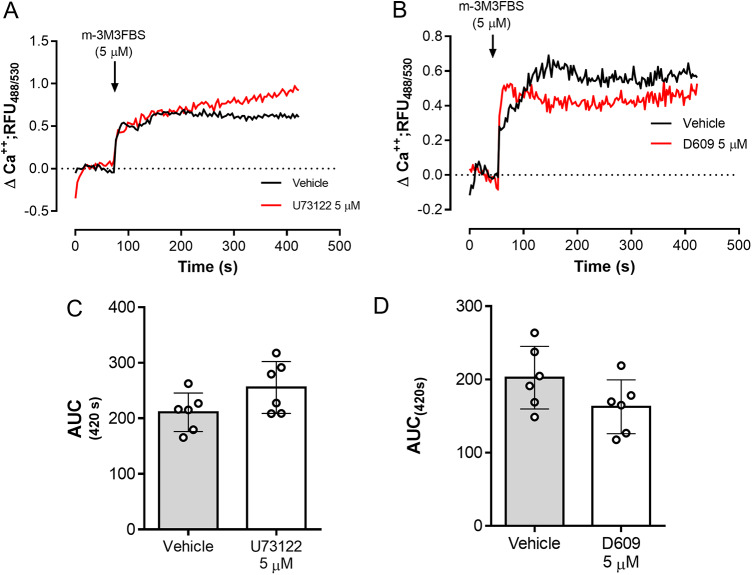
m-3M3FBS-driven Ca^2+^ flux in *B. besnoiti* tachyzoites is PLC-independent. Fluorometric registries and respective AUC of fluo-4-loaded *B. besnoiti* tachyzoites exposed to m-3M3FBS (5 µM) in the presence or absence of 5 µM U73122 (A, C) or 5 µM D609 (B, D). Bar graphs show the average values ± standard deviation of the AUC at 420 s of six biological replicates. **p ⩽ 0.01.

The role of PLC activation in tachyzoite-related Ca^2+^ flux was addressed by using the PLC-agonist m-3M3FBS (Bae et al. 2003). As illustrated in Figure 3A, m-3M3FBS tachyzoite treatments at 1.25-5 µM concentrations induced a dose-dependent Ca^2+^ flux over time. In addition, AUC analyses revealed an m-3M3FBS-driven, statistically significant increase in Ca^2+^ fluxes at 5 µM (*p* = 0.0014) (Figure 3A–B). EC_50_ calculation stated an effective concentration of 24.7 ± 1.5 µM (Supplementary Figure 1A). Notably, prolonged exposure (240 s) to 25 µM m-3M3FBS concentration also affected tachyzoite morphological integrity as evidenced by live cell 3D-holotomographic microscopy (Supplementary Figure 1B). Finally, to confirm m-3M3FBS specificity in *B. besnoiti*, we performed functional analyses on fluo-4-loaded *B. besnoiti* tachyzoites either in the presence or absence of PLC inhibitors (U73122 or D609). Unexpectedly, both compounds failed to interfere with m-3M3FBS-driven Ca^2+^ flux kinetics or amplitude over time, thereby indicating PLC-independent effects of m-3M3FBS (Figure 4A–B). This observation was quantitatively confirmed by AUC analyses, where no statistically significant differences were found when comparing vehicle or PLC inhibitor-treated tachyzoites (Figure 4C–D).

## Discussion

During acute merogonic replication cycles, coccidian parasites like *B. besnoiti* must actively invade, proliferate and egress from suitable host cells (Black and Boothroyd, 2000). In this context, cumulative evidence from *T. gondii*- and *P. falciparum*-related data indicates a pivotal role of Ca^2+^-dependent routes governing these processes in apicomplexan parasites (Lourido and Moreno, 2015). Since intracellular parasitism implies a close interaction of two eukaryotic cells, the maintenance of different Ca^2+^ concentrations in both cell types represents a challenging scenario *in vivo*. To address this, we performed an analysis on *B. besnoiti*-infected primary bovine endothelial host cells to be as close as possible to the *in vivo* situation (Alvarez-García et al. 2013). Current data revealed that at 24 h p.i., infected BUVEC layers show higher fluo-4-driven fluorescent signals than earlier infection time points (i.e. 6 and 12 h p.i.), suggesting that parasite intracellular proliferation is indeed directly linked to increased Ca^2+^ concentration in infected cell layers. In line, Velásquez et al. (2020) showed that after 6 and 12 h p.i. most BUVEC still contain one and two tachyzoites, respectively, whilst full proliferation of the parasite is achieved at 24 h p.i., leading to the formation of 8–16 tachyzoites/host cell thereby implying that Ca^2+^-derived fluorescent signal increase indeed is consequence of tachyzoite replication. Current data are in line with findings on *N. caninum*-infected BUVEC at 24 and 42 h p.i. (Larrazabal et al. 2021a), where 3D-holotomography revealed that Ca^2+^-derived signals accumulated within the intracellular meront and that intracellular *N. caninum* tachyzoites were the principal source of Ca^2+^ signals. Likewise, enhanced Ca^2+^ levels in meronts (72% higher than in host cytoplasm) were detected in *T. gondii*-infected human epidermoid carcinoma epithelial cells at 24 and 48 h p.i. (Pingret et al. 1996). These findings suggest that *B. besnoiti* tachyzoites infecting BUVECs should have a similar Ca^2+^ distribution in time points later than 24 h p.i. Referring to general cytoplasmic Ca^2+^ levels, apicomplexans like *T. gondii, P. falciparum* or *P. chabaudi* share typical concentrations with other eukaryotic cells by 70–100 nM (Garcia et al. 1996; Moreno and Zhong, 1996).

During the coccidian lytic cycle, free-released tachyzoites must actively invade new suitable host cells, which support the parasite’s metabolic requirements (Black and Boothroyd, 2000). In this context, PLC-driven signalling is considered essential for host cell invasion by apicomplexans. Nonetheless, considering the absence of IP3R or RyR homologues in these protozoan parasites, the participation of this route in apicomplexans remains unclear (Garcia et al. 2017). To gain functional insights into PLC-dependent pathways in *B. besnoiti* tachyzoites, we performed cell invasion assays after pre-treating tachyzoites with the PLC inhibitors U17122 and D609. The infective capacity of tachyzoites was estimated based on infection rates at 1 and 3 h p.i. Our findings indicate that both compounds interfere with the host cell infection, suggesting the involvement of a PLC-dependent pathway in *B. besnoiti* tachyzoite host cell invasion. However, considering the known off-target effects of U73122 on SERCA in other cell models (Taylor and Broad, 1998; Macmillan and McCarron, 2010), the observed impact of this compound on the invasion step may also involve PLC-independent mechanisms. Referring to compound D609, its anti-invasive effect was already reported for *T. gondii* tachyzoites, where respective treatments reduced cell invasion of human MRC5 fibroblasts by 69% but at a considerably higher concentration (100 µM) (Ricard et al. 1999).

Physiologically, PLC activation triggers cytoplasmic Ca^2+^ release from intracellular stores, depending on IP_3_ receptor activation (Berridge, 2016). In non-excitable cells like endothelial cells, this early event of Ca^2+^ flux promotes the opening of cytoplasmic Ca^2+^ channels, thereby allowing for extracellular Ca^2+^entry in a process named store-operated Ca^2+^ entry (SOCE) (Hogan and Rao, 2015). Referring to this pathway, we explored the role of intra- and extracellular Ca^2+^ sources for *B. besnoiti* tachyzoite invasion by using treatments with BAPTA and EGTA, serving as selective chelators of intra- and extracellular Ca^2+^, respectively. Current data show that intracellular Ca^2+^ chelation results in a marked reduction of *B. besnoiti* infective capacity. In contrast, extracellular Ca^2+^ chelation by EGTA treatments (5 mM) had no statistically significant effects on infection rates (20.62 ± 7.80% reduction), without inducing any significant effect in BAPTA-treated tachyzoites. Overall, these findings strongly suggest that *B. besnoiti* tachyzoites mainly rely on intracellular Ca^2+^ stores to actively invade host cells. In agreement, the pivotal role of intracellular Ca^2+^ for parasite invasion was also documented for *T. gondii* tachyzoites by BAPTA treatments (Lovett et al. 2002; Song et al. 2004; Del Carmen et al. 2009). However, the role of extracellular Ca^2+^ entry during the apicomplexa invasive process remains unclear. In case of *T. gondii*, EGTA pre-treatments of tachyzoites at double concentration (10 mM) dampened parasite invasion into HeLa cells at 4 and 18 h p.i. (Song et al. 2004). However, the capacity of EGTA to affect invasion seemed to be Ca^2+^-independent since *T. gondii* infectivity was shown to be mainly affected by EGTA-driven extracellular acidification (Lovett and Sibley, 2003). This last indicates that further studies are necessary to fully establish the participation of extracellular Ca^2+^ sources in *B. besnoiti* invasion.

Even though PLC signalling appears highly crucial in apicomplexan parasites, so far, physiological ligands capable of activating this pathway in resting tachyzoites have not been identified. In this report, we characterised Ca^2+^ fluxes induced by the only reported pharmacological PLC activator m-3M3FBS (Bae et al. 2003). Since – to our knowledge – no information is available on the suitable working concentration of m-3M3FBS for protozoan parasites, we first estimated the respective EC_50_ concentration (24.7 µM) using Ca^2+^-driven signals as readout. Of note, 25 µM m-3M3FBS induced cytotoxic effects in *B. besnoiti* tachyzoites at 3 min post-exposure. However, when applying 5 µM m-3M3FBS, this detrimental effect was absent and respective treatments led to a fast and sustained Ca^2+^ flux in *B. besnoiti* tachyzoites, confirming the principal functionality of this compound in these parasite stages. However, when assessing PLC-dependency of m-3M3FBS-driven Ca^2+^ fluxes by registering Ca^2+^ fluxes in the additional presence of the PLC inhibitors U17122 and D609, current data revealed that m-3M3FBS-driven Ca^2+^ flux did not change and therefore seemed PLC-independent. Overall, m-3M3FBS-driven, PLC-independent Ca^2+^ fluxes were already reported in human neuroblastoma (Krjukova et al. 2004). Furthermore, other PLC-independent effects of m-3M3FBS, such as apoptosis in the pro-monocytic human cell line U937 (Lee et al. 2005), suggested that data resulting from the use of this compound as PLC agonist must be interpreted with caution and need further investigations.

In summary, we here observed that *B. besnoiti* tachyzoites accumulate Ca^2+^ during their intracellular merogony and confirmed intracellular stores as the main Ca^2+^ sources during host cell invasion. Likewise, functional inhibition of cell invasion by PLC inhibitors suggests that this process is governed – at least partially – by a PLC-dependent mechanism. Finally, m-3M3FBS treatments induced a dose-dependent increase of Ca^2+^ flux, which was not affected by PLC inhibition in *B. besnoiti* tachyzoites. Overall, these novel data can be useful for the development of new therapeutic agents, targeting intracellular Ca^2+^ homeostasis and/or PLC-dependent signals in *B. besnoiti* tachyzoites, to control bovine besnoitiosis; nonetheless, further studies with a larger sample size and/or *in vivo* experiments are still necessary to establish the suitability of this therapeutic strategy. Likewise, the use of molecular tools, such as shRNA, CRISPR-Cas9 and transcriptomics/proteomics-derived data, represents a valuable opportunity to discover and validate novel targets involved in *B. besnoiti* Ca^2+^ homeostasis. In this context, elucidating the mechanisms underlying *B. besnoiti* Ca^2+^ homeostasis regulation during both acute and chronic infection, as well as exploring the parasite’s potential ability to modulate bovine host Ca^2+^ signalling, could provide novel insights into the biology of this neglected parasitosis.

## Supporting information

10.1017/S0031182025101182.sm001 Larrazabal et al. supplementary material Larrazabal et al. supplementary material

## Supplementary material

The supplementary material for this article can be found at https://doi.org/10.1017/S0031182025101182.

## References

[ref1] Alvarez-García G, Frey CF, Mora LMO and Schares G (2013) A century of bovine besnoitiosis: An unknown disease re-emerging in Europe. *Trends in Parasitology* 29, 407–415. 10.1016/j.pt.2013.06.002 23830145

[ref2] Alvarez-García G, García-Lunar P, Gutiérrez-Expósito D, Shkap V and Ortega-Mora LM (2014) Dynamics of *Besnoitia besnoiti* infection in cattle. *Parasitology* 141, 1419–1435. 10.1017/S0031182014000729 24871877

[ref3] Alves E, Bartlett PJ, Garcia CRS and Thomas AP (2011) Melatonin and IP3-induced Ca2+ release from intracellular stores in the malaria parasite *Plasmodium falciparum* within infected red blood cells. *The Journal of Biological Chemistry* 286, 5905–5912. 10.1074/jbc.M110.188474 21149448 PMC3037703

[ref4] Bae Y-S, Lee TG, Park JC, Hur JH, Kim Y, Heo K, Kwak J-Y, Suh P-G and Ryu SH (2003) Identification of a compound that directly stimulates phospholipase C activity. *Molecular Pharmacology* 63, 1043–1050. 10.1124/mol.63.5.1043 12695532

[ref5] Berridge MJ (2016) The Inositol Trisphosphate/Calcium signaling pathway in health and disease. *Physiological Reviews* 96, 1261–1296. 10.1152/physrev.00006.2016 27512009

[ref6] Black MW and Boothroyd JC (2000) Lytic cycle of Toxoplasma gondii. *Microbiology and Molecular Biology Reviews : MMBR* 64, 607–623. 10.1128/mmbr.64.3.607-623.2000 10974128 PMC99006

[ref7] Bullen HE, Jia Y, Yamaryo-Botté Y, Bisio H, Zhang O, Jemelin NK, Marq J-B, Carruthers V, Botté CY and Soldati-Favre D (2016) Phosphatidic acid-mediated signaling regulates microneme secretion in *toxoplasma*. *Cell Host & Microbe* 19, 349–360. 10.1016/j.chom.2016.02.006 26962945

[ref8] Burda P-C, Ramaprasad A, Bielfeld S, Pietsch E, Woitalla A, Söhnchen C, Singh MN, Strauss J, Sait A, Collinson LM, Schwudke D, Blackman MJ and Gilberger T-W (2023) Global analysis of putative phospholipases in *Plasmodium falciparum* reveals an essential role of the phosphoinositide-specific phospholipase C in parasite maturation. *mBio* 14, e0141323. 10.1128/mbio.01413-23 37489900 PMC10470789

[ref9] Carafoli E and Krebs J (2016) Why calcium? How calcium became the best communicator. *The Journal of Biological Chemistry* 291, 20849–20857. 10.1074/jbc.R116.735894 27462077 PMC5076498

[ref10] Del Carmen MG, Mondragón M, González S and Mondragón R (2009) Induction and regulation of conoid extrusion in *Toxoplasma gondii*. *Cellular Microbiology* 11, 967–982. 10.1111/j.1462-5822.2009.01304.x 19416276

[ref11] Fang J, Marchesini N and Moreno SNJ (2006) A *Toxoplasma gondii* phosphoinositide phospholipase C (*Tg* PI-PLC) with high affinity for phosphatidylinositol. *Biochemical Journal* 394, 417–425. 10.1042/BJ20051393 16288600 PMC1408672

[ref12] Garcia CR, Dluzewski AR, Catalani LH, Burting R, Hoyland J and Mason WT (1996) Calcium homeostasis in intraerythrocytic malaria parasites. *European Journal of Cell Biology* 71, 409–413. 8980913

[ref13] Garcia CRS, Alves E, Pereira PHS, Bartlett PJ, Thomas AP, Mikoshiba K, Plattner H and Sibley LD (2017) InsP3 signaling in apicomplexan parasites. *Current Topics in Medicinal Chemistry* 17, 2158–2165. 10.2174/1568026617666170130121042 28137231 PMC5490149

[ref14] Hogan PG and Rao A (2015) Store-operated calcium entry: mechanisms and modulation. *Biochemical and Biophysical Research Communications* 460, 40–49. 10.1016/j.bbrc.2015.02.110 25998732 PMC4441756

[ref15] Hortua Triana MA, Márquez-Nogueras KM, Vella SA and Moreno SNJ (2018) Calcium signaling and the lytic cycle of the apicomplexan parasite *toxoplasma gondii. Biochimica et Biophysica Acta*. *Molecular Cell Research* 1865, 1846–1856. 10.1016/j.bbamcr.2018.08.004 30992126 PMC6477927

[ref16] Jiménez-Meléndez A, Fernández-Álvarez M, Calle A, Ramírez MÁ, Diezma-Díaz C, Vázquez-Arbaizar P, Ortega-Mora LM and Álvarez-García G (2019) Lytic cycle of *Besnoitia besnoiti* tachyzoites displays similar features in primary bovine endothelial cells and fibroblasts. *Parasites and Vectors* 12, 517. 10.1186/s13071-019-3777-0 31685001 PMC6829937

[ref17] Jiménez-Meléndez A, Ojo KK, Wallace AM, Smith TR, Hemphill A, Balmer V, Regidor-Cerrillo J, Ortega-Mora LM, Hehl AB, Fan E, Maly DJ, Van Voorhis WC and Álvarez-García G (2017) *In vitro* efficacy of bumped kinase inhibitors against *Besnoitia besnoiti* tachyzoites. *International Journal for Parasitology* 47, 811–821. 10.1016/j.ijpara.2017.08.005 28899692 PMC13252795

[ref18] Jiménez-Meléndez A, Ramakrishnan C, Hehl AB, Russo G and Álvarez-García G (2020) RNA-Seq analyses reveal that endothelial activation and fibrosis are induced early and progressively by *besnoitia besnoiti* host cell invasion and proliferation. *Frontiers in Cellular and Infection Microbiology* 10, 218. 10.3389/fcimb.2020.00218 32500038 PMC7242738

[ref19] Krjukova J, Holmqvist T, Danis AS, Akerman KEO and Kukkonen JP (2004) Phospholipase C activator m-3M3FBS affects Ca2+ homeostasis independently of phospholipase C activation. *British Journal of Pharmacology* 143, 3–7. 10.1038/sj.bjp.0705911 15302681 PMC1575272

[ref20] Langenmayer MC, Gollnick NS, Majzoub-Altweck M, Scharr JC, Schares G and Hermanns W (2015) Naturally acquired bovine besnoitiosis: Histological and immunohistochemical findings in acute, subacute, and chronic disease. *Veterinary Pathology* 52, 476–488. 10.1177/0300985814541705 25096291

[ref21] Larrazabal C, Hermosilla C, Taubert A and Conejeros I (2021a) 3D holotomographic monitoring of Ca(++) dynamics during ionophore-induced *Neospora caninum* tachyzoite egress from primary bovine host endothelial cells. *Parasitology Research* 121, 1169–1177. 10.1007/s00436-021-07260-2 34386856 PMC8986705

[ref22] Larrazabal C, López-Osorio S, Velásquez ZD, Hermosilla C, Taubert A and Silva LMR (2021b) Thiosemicarbazone copper chelator blt-1 blocks apicomplexan parasite replication by selective inhibition of scavenger receptor B type 1 (SR-BI). *Microorganisms* 9(11), 2372. 10.3390/microorganisms9112372 34835496 PMC8622581

[ref23] Lee Y-N, Lee H-Y, Kim J-S, Park C, Choi YH, Lee TG, Ryu SH, Kwak J-Y and Bae Y-S (2005) The novel phospholipase C activator, m-3M3FBS, induces monocytic leukemia cell apoptosis. *Cancer Letters* 222, 227–235. 10.1016/j.canlet.2004.09.017 15863272

[ref24] Lourido S and Moreno SNJ (2015) The calcium signaling toolkit of the Apicomplexan parasites *Toxoplasma gondii* and *Plasmodium* spp. *Cell Calcium* 57, 186–193. 10.1016/j.ceca.2014.12.010 25605521 PMC4428288

[ref25] Lovett JL, Marchesini N, Moreno SNJ and Sibley LD (2002) *Toxoplasma gondii* microneme secretion involves intracellular Ca(2+) release from inositol 1,4,5-triphosphate (IP(3))/ryanodine-sensitive stores. *The Journal of Biological Chemistry* 277, 25870–25876. 10.1074/jbc.M202553200 12011085

[ref26] Lovett JL and Sibley LD (2003) Intracellular calcium stores in *Toxoplasma gondii* govern invasion of host cells. *Journal of Cell Science* 116, 3009–3016. 10.1242/jcs.00596 12783987

[ref27] Macmillan D and McCarron JG (2010) The phospholipase C inhibitor U-73122 inhibits Ca(2+) release from the intracellular sarcoplasmic reticulum Ca(2+) store by inhibiting Ca(2+) pumps in smooth muscle. *British Journal of Pharmacology* 160, 1295–1301. 10.1111/j.1476-5381.2010.00771.x 20590621 PMC2938802

[ref28] Maksimov P, Hermosilla C, Kleinertz S, Hirzmann J and Taubert A (2016) *Besnoitia besnoiti* infections activate primary bovine endothelial cells and promote PMN adhesion and NET formation under physiological flow condition. *Parasitology Research* 115, 1991–2001. 10.1007/s00436-016-4941-5 26847631

[ref29] Moreno SN and Zhong L (1996) Acidocalcisomes in *Toxoplasma gondii* tachyzoites. *The Biochemical Journal* 313(Pt 2), 655–659. 10.1042/bj3130655 8573106 PMC1216957

[ref30] Passos AP and Garcia CR (1998) Inositol 1,4,5-trisphosphate induced Ca2+ release from chloroquine-sensitive and -insensitive intracellular stores in the intraerythrocytic stage of the malaria parasite *P. chabaudi*. *Biochemical and Biophysical Research Communications* 245, 155–160. 10.1006/bbrc.1998.8338 9535800

[ref31] Pingret L, Millot JM, Sharonov S, Bonhomme A, Manfait M and Pinon JM (1996) Relationship between intracellular free calcium concentrations and the intracellular development of *Toxoplasma gondii*. *The Journal of Histochemistry and Cytochemistry: Official Journal of the Histochemistry Society* 44, 1123–1129. 10.1177/44.10.8813077 8813077

[ref32] Ricard J, Pelloux H, Favier AL, Gross U, Brambilla E and Ambroise-Thomas P (1999) *Toxoplasma gondii*: Role of the phosphatidylcholine-specific phospholipase C during cell invasion and intracellular development. *Experimental Parasitology* 91, 231–237. 10.1006/expr.1998.4353 10072325

[ref33] Song H-O, Ahn M-H, Ryu J-S, Min D-Y, Joo K-H and Lee Y-H (2004) Influence of calcium ion on host cell invasion and intracellular replication by Toxoplasma gondii. *The Korean Journal of Parasitology* 42, 185–193. 10.3347/kjp.2004.42.4.185 15591836 PMC2717384

[ref34] Taubert A, Zahner H and Hermosilla C (2006) Dynamics of transcription of immunomodulatory genes in endothelial cells infected with different coccidian parasites. *Veterinary Parasitology* 142, 214–222. 10.1016/j.vetpar.2006.07.021 16930845

[ref35] Taylor CW and Broad LM (1998) Pharmacological analysis of intracellular Ca2+ signalling: Problems and pitfalls. *Trends in Pharmacological Sciences* 19, 370–375. 10.1016/s0165-6147(98)01243-7 9786025

[ref36] Velásquez ZD, Lopez-Osorio S, Pervizaj-Oruqaj L, Herold S, Hermosilla C and Taubert A (2020) *Besnoitia besnoiti*-driven endothelial host cell cycle alteration. *Parasitology Research* 119, 2563–2577. 10.1007/s00436-020-06744-x 32548739 PMC7366594

