# Supplementary material for: Functional analysis of Ca2+ signalling in Besnoitia besnoiti tachyzoites
Source: Parasitology. 2025 Nov 10;153(1):35–42. doi: 10.1017/S0031182025101182 (PMC13215729; doi:10.1017/S0031182025101182)
Supplement: Larrazabal et al. supplementary material [file S0031182025101182sup001.zip › S0031182025101182sup001/Supplementary_table.pdf]

Supplementary Table 1. Descriptive statistics from the main experiments performed in this work

Assay	Biol. replicates	Readout	Experimental conditions	Mean	S.D.	CI of the mean (95%)	25 % percentile	Median	75 % percentile
Ca^{2+} changes in *B. besnoiti* infected monolayer	5	Normalized fluorescence intensity	6 h p.i.	0.94	0.05	0.88 - 0.99	0.90	0.91	0.97
			12 h p.i.	0.97	0.05	0.92 - 1.02	0.94	0.96	1.0
			24 h p.i.	1.25	0.54	1.19 - 1.31	1.20	1.25	1.30
Participation PLC during *B. besnoiti* infection	6	Infection Rate	Vehicle (1 h p.i.)	20.72	3.08	17.49 - 23.95	18.50	20.17	23.00
			U73122 (1 h p.i.)	6.44	3.23	3.06 - 9.83	3.92	5.67	9.20
			Vehicle (1 h p.i.)	26.67	5.89	20.49 - 32.85	22.00	26.00	31.50
			D609 (1 h p.i.)	17.38	5.38	11.74 - 23.03	12.92	15.83	23.41
			Vehicle (3 h p.i.)	26.56	6.85	19.37 - 33.74	19.83	26.00	33.17
			U73122 (3 h p.i.)	5.00	1.19	3.75 - 6.25	4.17	4.67	6.00
			Vehicle (3 h p.i.)	38.44	1.72	36.64 - 40.25	37.00	38.33	40.17
			D609 (3 h p.i.)	19.39	3.92	15.27 - 23.51	17.17	18.83	22.17
Participation of intracellular and extracellular Ca^{2+} sources during *B. besnoiti* infection	6	Infection Rate	Vehicle (1 h p.i.)	15.00	2.62	12.25 - 17.75	12.58	14.67	17.08
			BAPTA (1 h p.i.)	4.00	1.10	2.85 - 5.15	3.250	4.33	4.75
			EGTA (1 h p.i.)	11.83	1.99	9.74 - 13.93	10.17	11.33	13.75
			B+E (1 h p.i.)	3.50	0.91	2.54 - 4.46	2.67	3.33	4.25
			Vehicle (3 h p.i.)	19.72	3.14	16.43 - 23.01	17.83	20.17	21.50
			BAPTA (3 h p.i.)	2.78	1.67	1.03 - 4.53	1.33	11.67	4.00
			EGTA (3 h p.i.)	11.94	2.06	9.78 - 14.11	10.33	3.00	13.58
			B+E (3 h p.i.)	1.06	0.68	0.34 - 1.77	0.50	1.67	1.50

Characterization of m-3M3FBS-induced Ca^{2+} fluxes	6	Area under the curve analysis	Vehicle	96.83	35.27	59.82 - 133.8	58.83	99.95	125.9
			1.25 µM	126.3	27.09	97.84 - 154.7	110.2	114.4	143.4
			2.5 µM	160.5	37.95	120.7 - 200.4	136.0	158.9	196.9
			5 µM	264.3	79.45	180.9 - 347.6	189.4	295.9	324.1
Role of PLC on m-3M3FBS-induced Ca^{2+} fluxes	6	Area under the curve analysis	Vehicle	211.0	34.65	174.6 - 247.3	176.1	215.7	235.8
			U73122	255.6	46.65	206.6 - 304.5	208.8	253.5	298.0
			Vehicle	202.4	202.4	157.5 - 247.3	163.7	197.9	244.2
			D609	162.6	162.6	124.0 - 201.2	124.4	167.2	188.4
