# Supplementary figures and images for: Functional analysis of Ca2+ signalling in Besnoitia besnoiti tachyzoites
Source: Parasitology. 2025 Nov 10;153(1):35–42. doi: 10.1017/S0031182025101182 (PMC13215729; doi:10.1017/S0031182025101182)

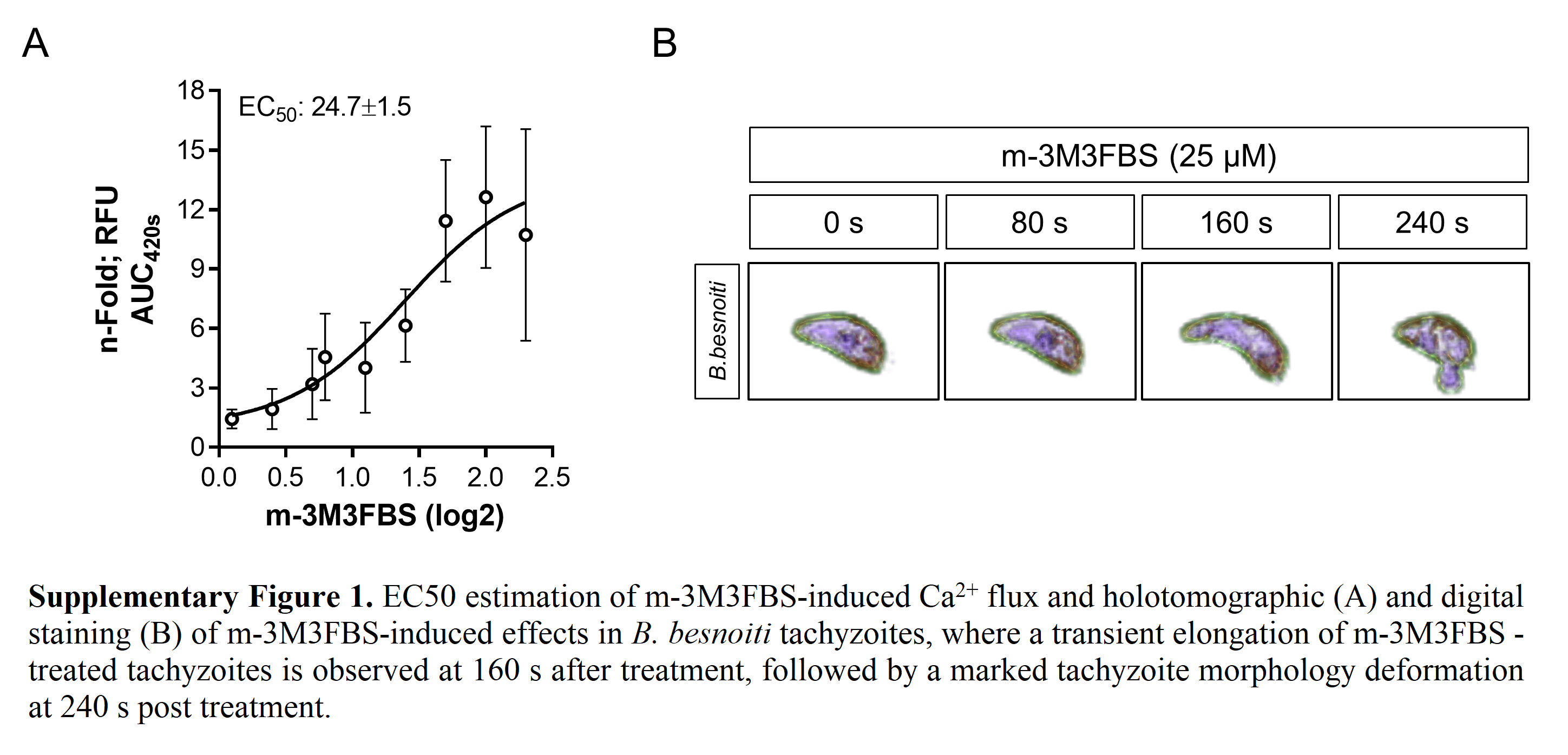

Supplement: Larrazabal et al. supplementary material [file S0031182025101182sup001.zip › S0031182025101182sup001/Supplementary Figure 1.tif]

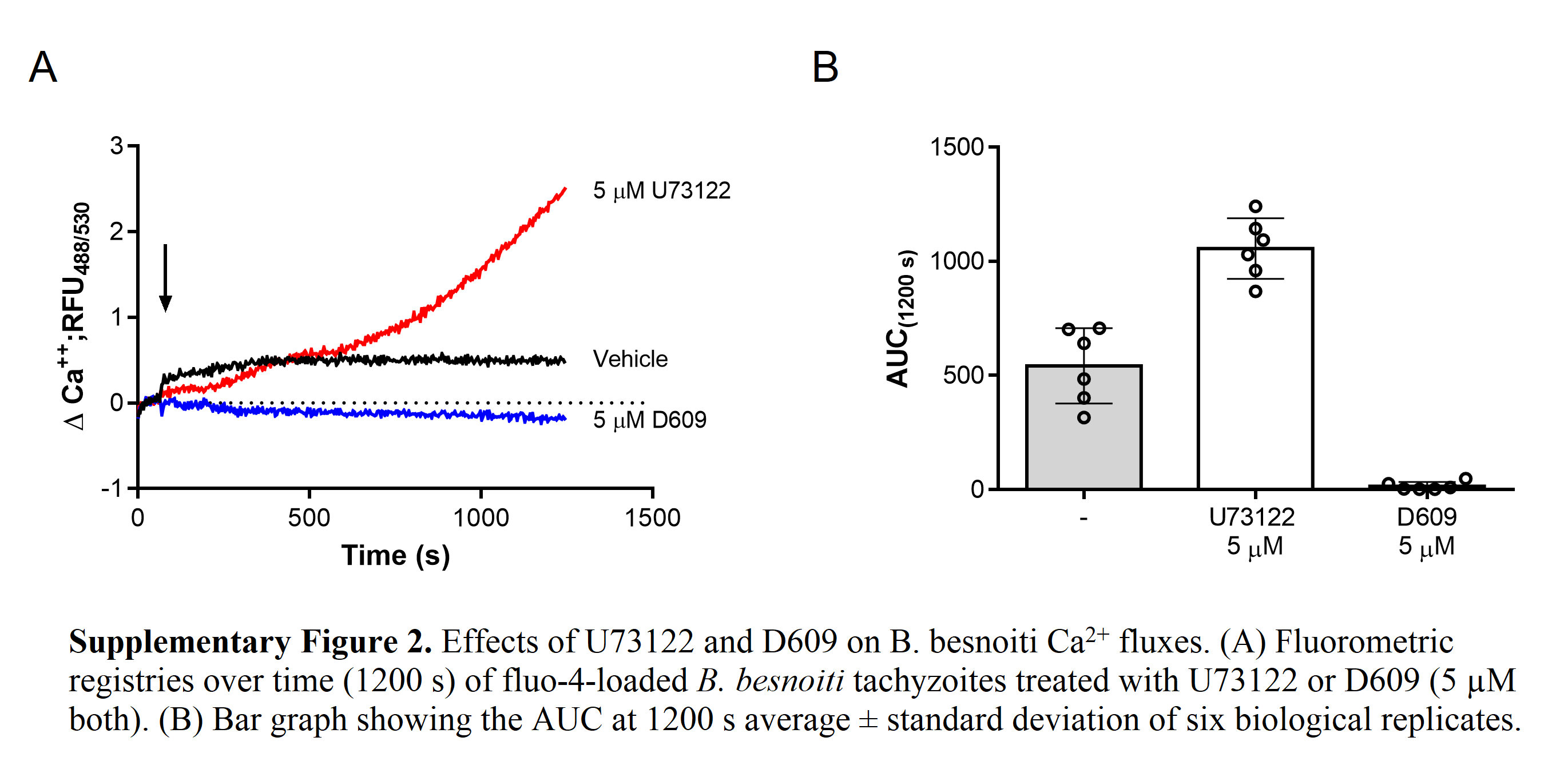

Supplement: Larrazabal et al. supplementary material [file S0031182025101182sup001.zip › S0031182025101182sup001/Supplementary Figure 2.tif]
